# Case of Chronic Cystitis, Terminating Fatally

**Published:** 1842-01

**Authors:** R. B. Harper

**Affiliations:** Tipton county, Tennessee


**Art. V.**—*Case of Chronic Cystitis, terminating fatally.* By Dr. R. B. Harper, of Tipton county, Tennessee.

The subject of this case was a boy 3 years of age, the son of N. H. N. of this county. The history of the case as given by the parents was as follows: Some time during the latter end of last autumn, their son told them that a rail had fallen on him, but it gave them little uneasiness as he did not complain of much pain. The first symptom that excited alarm was the passage with his urine of a considerable quantity of blood. This discharge kept up for several days, becoming mingled with what appeared to be pus—sometimes with long white lumps, as his parents expressed it, which evidently was coagulated lymph. This symptom occurred early in January. After a time the lymph ceased to be discharged and he began to experience great difficulty in voiding his urine. The usual preparation of balsam copaiva was prescribed by a physician, but without any advantage. The urine was voided drop by drop, and accumulating in the bladder gave him great distress. I was applied to on the 19th of March, and on examination found the bladder much distended, the external orifice of the urethra inflamed, the desire to micturate incessant, and the discharge of urine which was scant attended with severe pain.

I prescribed an ointment which removed the inflammation of the external orifice of the urethra, and relieved the pain of micturition. Ordered cathartics, blisters and uva ursi, which generally afford relief in such cases, but in this without benefit, as the bladder continued to be distended. I resorted to the use of the catheter and pressure on the abdomen, but with no better results. The urine did not flow through the catheter, and yet I was satisfied that there was no mechanical obstruction, and that the difficulty grew out of a deposit of coagulable lymph in the bladder which had nearly filled its cavity. This was verified by the autopsy, which was afforded by the death of the patient, inflammation of the brain having supervened and terminated his sufferings on the 20th of April.

*Autopsy.* An incision having been made above the pubis into the bladder it discovered that it was nearly filled with coagulated lymph, amounting to about half a pint in quantity. It had formed an attachment to the bladder on its surface between the ureters. A portion of the lymph was pendulous, and extended into the neck of the bladder. That portion was red and apparently organized, resembling the heart of a squirrel. Other portions were white and fibrous in their texture with bloodvessels passing through their substance. I have preserved the specimen for the Pathological Cabinet of the Medical Institute of Louisville.

I will subjoin a memorandum of a case of *ovarian dropsy,* in which I resorted to tapping with success.

Mrs. F., of Obion county, æt. 35 years, was the subject of this disease, which had existed about 10 years. I resorted to an operation on the 12th of May, 1840, and drew off four gallons of water. The fluid has not collected again, and the lady now enjoys excellent health.

May 4, 1841.

